# Triple islet autoantibody–positive type 1 diabetes in Japanese stiff-person syndrome despite a protective HLA haplotype

**DOI:** 10.1210/jcemcr/luag154

**Published:** 2026-06-09

**Authors:** Masatoshi Suenaga, Kazuma Ogiso, Naoya Takeuchi, Hiroki Akahoshi, Yoshihiko Nishio, Katsutaro Morino

**Affiliations:** Department of Diabetes and Endocrine Medicine, Kagoshima University Graduate School of Medical and Dental Sciences, Kagoshima 890-8520, Japan; Department of Diabetes and Endocrine Medicine, Kagoshima University Graduate School of Medical and Dental Sciences, Kagoshima 890-8520, Japan; Department of Diabetes and Endocrine Medicine, Kagoshima University Graduate School of Medical and Dental Sciences, Kagoshima 890-8520, Japan; Department of Diabetes and Endocrine Medicine, Kagoshima University Graduate School of Medical and Dental Sciences, Kagoshima 890-8520, Japan; Department of Diabetes and Endocrine Medicine, Kagoshima University Graduate School of Medical and Dental Sciences, Kagoshima 890-8520, Japan; Department of Diabetes and Endocrine Medicine, National Hospital Organization Kagoshima Medical Center, Kagoshima 892-0853, Japan; Department of Diabetes and Endocrine Medicine, Kagoshima University Graduate School of Medical and Dental Sciences, Kagoshima 890-8520, Japan

**Keywords:** stiff-person syndrome, autoimmune diabetes, islet autoantibodies, HLA haplotypes, beta-cell failure, GAD65 autoimmunity

## Abstract

Type 1 diabetes is a recognized complication of stiff-person syndrome (SPS), yet its immunogenetic determinants in non-European populations remain poorly characterized. A 39-year-old Japanese woman with a 14-year history of SPS presented with diabetic ketoacidosis and was found to have autoimmune type 1 diabetes with rapid beta-cell loss despite long-term immunosuppression. Anti-GAD65 titers were persistently ≥2000 U/mL by enzyme immunoassay, with anti-IA-2 at 8.2 U/mL and anti-ZnT8 at 53.1 U/mL. Urinary C-peptide declined from 4.16 μg/day (SI: 1.38 nmol/day) to <0.9 μg/day (SI: <0.3 nmol/day) over 13 months, indicating rapid progression to near-complete beta-cell failure. High-resolution human leukocyte antigen (HLA) typing revealed a susceptibility haplotype DRB1*04:05-DQA1*03:03-DQB1*04:01, a protective haplotype DRB1*15:02-DQA1*01:03-DQB1*06:01, and HLA-A*24:02; DRB1*03:01, the allele most strongly associated with SPS in European cohorts, was absent. This case demonstrates that SPS-associated autoimmunity can override protective HLA haplotypes, resulting in triple islet autoantibody–positive type 1 diabetes with an immunogenetic profile distinct from that reported in European cohorts.

## Introduction

Stiff-person syndrome (SPS) is a rare autoimmune neurological disorder characterized by progressive truncal and limb rigidity, episodic painful spasms, and elevated serum anti-GAD65 antibodies [1]. Type 1 diabetes develops in approximately 30-40% of patients with SPS, typically presenting with isolated anti-GAD65 positivity and a slowly progressive course [2, 3].

The link between SPS and type 1 diabetes is largely attributed to GAD65, which is expressed in both pancreatic beta cells and GABAergic neurons [4, 5]. Despite this shared antigen, SPS-associated type 1 diabetes is considered immunologically distinct from classical autoimmune type 1 diabetes. Prior studies report that SPS-associated diabetes typically shows isolated anti-GAD65 positivity, with other islet autoantibodies such as anti-IA-2 and anti-ZnT8 infrequently detected [6]. In a cohort of 79 GAD65-seropositive patients with SPS (43% with type 1 diabetes), only 3 (4%) were IA-2 antibody-positive [6], suggesting limited epitope spreading beyond GAD65.

In European and North American cohorts, SPS-associated type 1 diabetes is strongly linked to the human leukocyte antigen (HLA) haplotype DRB1*03:01-DQB1*02:01 [4, 7], yet the immunogenetic architecture of SPS in Asian patients, particularly in Japan, remains poorly characterized. Reports of SPS-associated type 1 diabetes from Japan are limited. This case is notable for 2 reasons: first, simultaneous positivity for 3 islet autoantibodies (anti-GAD65, anti-IA-2, and anti-ZnT8) in a patient with SPS, a profile rarely described in the SPS-associated type 1 diabetes literature; second, a population-specific HLA pattern characterized by DRB1*04:05 in the absence of DRB1*03:01 despite a coexisting protective haplotype. These findings challenge assumptions derived from European cohorts and underscore the need for population-specific metabolic surveillance in SPS.

## Case presentation

A 39-year-old Japanese woman presented with her second episode of diabetic ketoacidosis (DKA). She had been diagnosed with SPS 14 years earlier, at age 25 years, following progressive truncal stiffness, painful lower-limb spasms, and gait disturbance; serum anti-GAD65 was 9820 U/mL by radioimmunoassay. Electromyography demonstrated continuous motor unit activity with co-contraction of antagonist muscles, and brain and spinal magnetic resonance imaging showed no structural abnormalities. Her family history was notable for Fisher syndrome, an autoimmune neuropathy, in her grandfather; no additional autoimmune diseases were reported in other family members. Long-term SPS therapy included intravenous methylprednisolone pulses, administered monthly from 2010 and reduced to every 2-3 months from 2016, and azathioprine, 50 mg every other day, added in 2016. Owing to refractory neurological symptoms, azathioprine was temporarily replaced by tacrolimus, 3 mg/day, from 2021 to 2023, but azathioprine was reinstated in 2023 because of insufficient neurological response.

Dysglycemia, initially attributed to corticosteroid exposure, developed gradually from a normal baseline, with hemoglobin A1c (HbA1c) 5.8% (SI: 40 mmol/mol) (reference range, 4.6%-6.2% [SI: 27-44 mmol/mol]) in January 2019. It was first documented in June 2019, when HbA1c was 6.4% (SI: 46 mmol/mol) and fasting plasma glucose was 124 mg/dL (SI: 6.9 mmol/L). By March 2020, HbA1c had increased to 6.5% (SI: 48 mmol/mol), with a 2-hour postprandial plasma glucose of 145 mg/dL (SI: 8.1 mmol/L), without osmotic or other symptoms suggestive of diabetes. Diabetes was first recognized in September 2020, when HbA1c reached 13.5% (SI: 124 mmol/mol) and random plasma glucose was 422 mg/dL (SI: 23.4 mmol/L), and insulin therapy was initiated.

**Figure 1:**
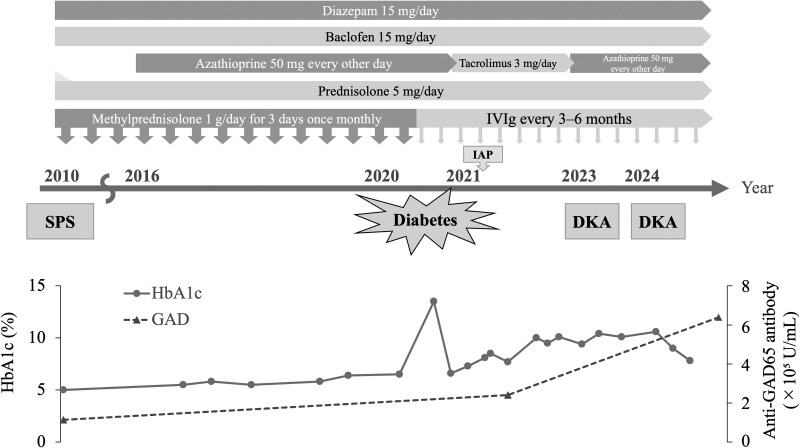
Clinical course of stiff-person syndrome and development of autoimmune type 1 diabetes. Clinical timeline showing the 14-year progression from SPS diagnosis in 2010 to autoimmune type 1 diabetes established at the index admission in 2024. Anti-GAD65 levels (dashed line, right y-axis) remained markedly elevated despite immunosuppressive therapy; the initial value of 9820 U/mL (2010) was measured by RIA, whereas subsequent values of 241 000 U/mL (2021) and 639 000 U/mL (2024) were obtained by EIA after the assay transition in 2015; therefore, RIA and EIA values are not directly comparable. HbA1c (solid line, left y-axis) rose progressively from 2019, with overt diabetes recognized in September 2020 (HbA1c 13.5%, SI: 124 mmol/mol), at which time insulin therapy was initiated. Two DKA episodes (February 2023 and March 2024) were each precipitated by insulin self-discontinuation. Triple islet autoantibody positivity (anti-GAD65, anti-IA-2, and anti-ZnT8) was confirmed in March 2024. Symptomatic SPS therapy comprised diazepam (15 mg/day) and baclofen (15 mg/day). Immunosuppressive therapy included oral prednisolone (5 mg/day), methylprednisolone pulses (1 g/course), intravenous immunoglobulin, immunoadsorption plasmapheresis (October 2021), and azathioprine (from 2016), with temporary substitution by tacrolimus from 2021 to 2023 for refractory neurological symptoms. Despite sustained combined therapy, progressive beta-cell failure continued. Abbreviations: DKA, diabetic ketoacidosis; EIA, enzyme immunoassay; GAD65, glutamic acid decarboxylase 65-kDa isoform; HbA1c, glycated hemoglobin; IAP, immunoadsorption plasmapheresis; IVIG, intravenous immunoglobulin; RIA, radioimmunoassay; SPS, stiff-person syndrome.

A first episode of DKA occurred in February 2023, 13 months before the index admission, precipitated by self-discontinuation of insulin; urinary C-peptide was 4.16 μg/day (SI: 1.38 nmol/day) (reference range, 29.2-167.0 μg/day [SI: 9.7-55.4 nmol/day]), indicating residual but declining beta-cell function (Fig. 1). The current episode was likewise precipitated by self-discontinuation of insulin.

## Diagnostic assessment

At the index admission in March 2024, autoimmune type 1 diabetes was established on the basis of triple islet autoantibody positivity and near-complete beta-cell loss, with HLA findings supporting autoimmune susceptibility.

On examination, the patient was alert and hemodynamically stable, with blood pressure 122/74 mmHg, heart rate 98 beats/min, respiratory rate 22 breaths/min, and temperature 36.7 °C. Respirations were deep and labored, consistent with Kussmaul respirations. Neurological examination revealed marked truncal rigidity with stimulus-evoked lower-limb spasms consistent with SPS activity.

Biochemical evaluation confirmed DKA: plasma glucose 581 mg/dL (SI: 32.2 mmol/L) (reference range, 70-110 mg/dL [SI: 3.9-6.1 mmol/L]), arterial pH 7.25 (reference range, 7.35-7.45), elevated anion gap 23.9 mmol/L (reference range, 8-16 mmol/L), and HbA1c 9.0% (SI: 75 mmol/mol) (Table 1). Fasting serum C-peptide was <0.03 ng/mL (SI: <0.01 nmol/L) (reference range, 0.80-2.50 ng/mL [SI: 0.27-0.83 nmol/L]) and urinary C-peptide was <0.9 μg/day (SI: <0.3 nmol/day), indicating near-complete loss of endogenous insulin secretion over the preceding 13 months, from 4.16 μg/day (SI: 1.38 nmol/day) at the first DKA episode. Both measurements were obtained during insulin-omission DKA, supporting severe endogenous insulin deficiency. A comprehensive islet autoantibody panel demonstrated anti-GAD65 ≥ 2000 U/mL, anti-IA-2 8.2 U/mL, and anti-ZnT8 53.1 U/mL (Table 2).

**Table 1:** Laboratory findings at DKA presentation (index admission, March 2024)

Parameter	Value	Reference range
Metabolic parameters		
Plasma glucose	581 mg/dL (SI: 32.2 mmol/L)	70-110 mg/dL (SI: 3.9-6.1 mmol/L)
HbA1c	9.0% (SI: 75 mmol/mol)	4.6-6.2% (SI: 27-44 mmol/mol)
Arterial pH	7.25	7.35-7.45
HCO_3_^−^	13.1 mmol/L	22-26 mmol/L
Anion gap	23.9 mmol/L	8-16 mmol/L
Ketone bodies (venous)		
Acetoacetate	2091 μmol/L	<55 μmol/L
3-Hydroxybutyrate	8740 μmol/L	<76 μmol/L
Urinalysis		
Glucose	4+	Negative
Ketone	4+	Negative
Specific gravity	1.030	1.010-1.025
Electrolytes		
Sodium	129 mmol/L	136-145 mmol/L
Potassium	5.3 mmol/L	3.5-5.0 mmol/L
Chloride	92 mmol/L	98-108 mmol/L
Osmolality	302 mOsm/kg	275-295 mOsm/kg
Renal function		
BUN	28.2 mg/dL (SI: 10.1 mmol/L)	8-20 mg/dL (SI: 2.9-7.1 mmol/L)
Creatinine	0.92 mg/dL (SI: 81 μmol/L)	0.46-0.79 mg/dL (SI: 41-70 μmol/L)
Uric acid	10.0 mg/dL (SI: 595 μmol/L)	2.5-7.0 mg/dL (SI: 149-416 μmol/L)
eGFR	49.5 mL/min/1.73 m^2^	>60 mL/min/1.73 m^2^
Hematology		
WBC	14 640/μL (SI: 14.64 × 10^9^/L)	3300-8600/μL (SI: 3.3-8.6 × 10^9^/L)
RBC	4.77 × 10^6^/μL (SI: 4.77 × 10^12^/L)	3.76-5.00 × 10^6^/μL (SI: 3.76-5.00 × 10^12^/L)
Hemoglobin	15.0 g/dL (SI: 150 g/L)	11.6-14.8 g/dL (SI: 116-148 g/L)
Hematocrit	43.5% (SI: 0.435)	35.1-44.4% (SI: 0.351-0.444)
Platelets	257 × 10^3^/μL (SI: 257 × 10^9^/L)	158-348 × 10^3^/μL (SI: 158-348 × 10^9^/L)
Hepatic function		
AST	21 U/L (SI: 0.35 μkat/L)	13-30 U/L (SI: 0.22-0.50 μkat/L)
ALT	22 U/L (SI: 0.37 μkat/L)	7-23 U/L (SI: 0.12-0.38 μkat/L)
Inflammation		
CRP	1.43 mg/dL (SI: 14.3 mg/L)	<0.14 mg/dL (SI: <1.4 mg/L)

Unit presentation and conversions: Values are presented in conventional units followed by SI units in parentheses, where applicable. Plasma glucose, HbA1c, BUN, creatinine, uric acid, hemoglobin, AST, ALT, and CRP are shown with SI equivalents in parentheses. Sodium, potassium, chloride, bicarbonate, and anion gap are reported in mmol/L, which is the routine clinical and SI-compatible unit in our setting; therefore, separate alternative units are not shown. Ketone body concentrations are reported in SI-derived units (μmol/L). Osmolality is presented in standard clinical units (mOsm/kg), and eGFR in mL/min/1.73 m^2^, consistent with routine clinical reporting. Arterial pH, urine dipstick results, and specific gravity are shown in standard clinical format and are not subject to SI conversion. Conversion factors were as follows: Glucose: mg/dL × 0.0555 = mmol/L; HbA1c: IFCC (mmol/mol) = (NGSP [%] − 2.15)/0.0915; BUN: mg/dL × 0.357 = mmol/L; Creatinine: mg/dL × 88.4 = μmol/L; Uric acid: mg/dL × 59.48 = μmol/L; Hemoglobin: g/dL × 10 = g/L; AST/ALT: U/L × 0.0167 = μkat/L; CRP: mg/dL × 10 = mg/L.

Abbreviations: ALT, alanine aminotransferase; AST, aspartate aminotransferase; BUN, blood urea nitrogen; CRP, C-reactive protein; DKA, diabetic ketoacidosis; eGFR, estimated glomerular filtration rate; HbA1c, glycated hemoglobin; RBC, red blood cell count; WBC, white blood cell count.

**Table 2:** Islet autoantibody profile and serial beta-cell function

Parameter	Time point	Value	Reference range
Anti-GAD65 antibody			
	July 2010 (SPS diagnosis)	9820 U/mL*^a^*	<5.0 U/mL
	October 2021	241 000 U/mL*^b^*	<5.0 U/mL
	March 2024 (DKA)	≥2000 U/mL*^c^*	<5.0 U/mL
	November 2024	639 000 U/mL*^b^*	<5.0 U/mL
Other islet autoantibodies			
Anti-IA-2 antibody	March 2024 (DKA)	8.2 U/mL	<0.6 U/mL
Anti-ZnT8 antibody	March 2024 (DKA)	53.1 U/mL	<15.0 U/mL
Beta-cell function			
Serum C-peptide			
	October 2021	0.10 ng/mL (SI: 0.033 nmol/L)	0.80-2.50 ng/mL (SI: 0.27-0.83 nmol/L)
	March 2024 (DKA) (fasting)*^d^*	<0.03 ng/mL (SI: <0.01 nmol/L)	0.80-2.50 ng/mL (SI: 0.27-0.83 nmol/L)
	March 2024 (DKA) (2 hours postprandial)*^e^*	0.04 ng/mL (SI: 0.013 nmol/L)	1.50-3.00 ng/mL (SI: 0.50-1.00 nmol/L)
Urinary C-peptide			
	February 2023	4.16 μg/day (SI: 1.38 nmol/day)	29.2-167.0 μg/day (SI: 9.7-55.4 nmol/day)
	March 2024 (DKA)	<0.9 μg/day (SI: <0.3 nmol/day)	29.2-167.0 μg/day (SI: 9.7-55.4 nmol/day)
	Decline over 13 months	>78%	NA

SI unit conversions: C-peptide (serum): ng/mL × 0.331 = nmol/L; C-peptide (urine): μg/day × 0.331 = nmol/day.

Abbreviations: DKA, diabetic ketoacidosis; GAD65, glutamic acid decarboxylase 65-kDa isoform; IA-2, insulinoma-associated antigen-2; SPS, stiff-person syndrome; ZnT8, zinc transporter 8.

^*a*^Measured by radioimmunoassay (RIA). The anti-GAD65 antibody assay was changed from RIA to enzyme immunoassay (EIA) in 2015; values obtained by different assay systems are not directly comparable.

^*b*^Measured by EIA after dilution.

^*c*^Measured by standard EIA; the upper limit of detection was 2000 U/mL.

^*d*^Plasma glucose at sampling: 190 mg/dL (10.5 mmol/L).

^*e*^Plasma glucose at sampling: 264 mg/dL (14.7 mmol/L).

The initial anti-GAD65 value of 9820 U/mL was measured by radioimmunoassay; the index-admission value of ≥2000 U/mL was obtained by a standard clinical enzyme immunoassay with an upper detection limit of 2000 U/mL. These assays are not directly comparable. High-resolution HLA typing identified a susceptibility haplotype, DRB1*04:05-DQA1*03:03-DQB1*04:01, and a protective haplotype, DRB1*15:02-DQA1*01:03-DQB1*06:01; HLA-A*24:02 was present, and DRB1*03:01 was absent (Table 3).

**Table 3:** High-resolution HLA typing results*^a^*

HLA locus	Allele 1	Allele 2	Significance
HLA-A	A*24:02:01	A*26:01:01	A*24:02 associated with complete beta-cell destruction*^b^*
HLA-B	B*52:01:01	B*52:01:01	Homozygous
HLA-C	C*08:01:01	C*12:02:02	NA
HLA-DRB1	DRB1*04:05:01	DRB1*15:02:01	Susceptibility/protective heterozygosity*^c^*
HLA-DQA1	DQA1*03:03:01	DQA1*01:03:01	NA
HLA-DQB1	DQB1*04:01:01	DQB1*06:01:01	NA
HLA-DRB3/4/5	DRB4*01:03:01	DRB5*01:02:01	NA

^*a*^All alleles were typed at three-field resolution using next-generation sequencing–sequence-based typing (NGS-SBT).

^*b*^HLA**-**A*24:02 has been associated with more complete beta-cell destruction and earlier insulin dependence in Japanese patients with type 1 diabetes [16, 17].

^*c*^The susceptibility haplotype was DRB1*04:05-DQA1*03:03-DQB1*04:01 [8], and the protective haplotype was DRB1*15:02-DQA1*01:03-DQB1*06:01 [8, 9]. The European SPS-associated haplotype DRB1*03:01-DQB1*02:01 (DR3-DQ2) was not detected [4, 7]. Haplotype phasing was inferred from established linkage disequilibrium patterns in Japanese populations [8, 9]. Direct three-field typing confirmed DQA1*01:03:01 (not DQA1*01:02), distinguishing this protective haplotype from its Caucasian counterpart DRB1*15:01-DQA1*01:02-DQB1*06:02 [10, 11].

Abbreviations: HLA, human leukocyte antigen; NGS-SBT, next-generation sequencing–sequence-based typing; SPS, stiff-person syndrome.

## Treatment

The patient's long-term SPS regimen comprised symptomatic neurological therapy with diazepam (15 mg/day) and baclofen (15 mg/day), and combination immunotherapy including oral prednisolone (5 mg/day), intravenous methylprednisolone pulses (1 g per course), intravenous immunoglobulin at established infusion intervals, and periodic immunoadsorption plasmapheresis, including a course in October 2021. Despite this sustained immunosuppressive regimen and the insulin therapy initiated in September 2020, beta-cell function continued to decline, culminating in the current DKA presentation.

At admission, DKA was managed with intravenous fluid resuscitation and continuous intravenous insulin infusion until anion gap closure. After resolution of ketoacidosis, the patient was transitioned to a basal-bolus insulin regimen (glargine once daily and aspart at mealtimes) with continuous glucose monitoring. The pre-existing SPS regimen was continued without modification.

## Outcome and follow-up

The patient was discharged in stable condition approximately 1 week after admission. At 3-month follow-up, HbA1c was 7.8% (SI: 62 mmol/mol) on the basal-bolus regimen, and no further DKA episodes had occurred. Neurological status was unchanged. Total follow-up from index admission was approximately 8 months.

At final follow-up in November 2024, serum anti-GAD65 had increased to 639 000 U/mL, compared with 241 000 U/mL measured in 2021 despite uninterrupted immunosuppression (Table 2). Both values were obtained with an extended-range enzyme immunoassay platform and are not directly comparable to the index-admission measurement of ≥2000 U/mL obtained by standard clinical enzyme immunoassay, suggesting persistent islet autoimmunity.

## Discussion

This case provides insights into the immunopathogenesis and genetic determinants of SPS-associated type 1 diabetes. The patient developed autoimmune type 1 diabetes with severe beta-cell failure and simultaneous positivity for multiple islet autoantibodies despite long-term immunosuppression and a generally protective HLA haplotype. The absence of HLA-DRB1*03:01, the allele most strongly associated with SPS in European populations, highlights population-specific genetic risk patterns.

SPS-associated type 1 diabetes is typically characterized by isolated anti-GAD65 positivity and a slowly progressive course [3, 6]. In contrast, this patient exhibited concurrent anti-GAD65, anti-IA-2, and anti-ZnT8 antibodies. In broader type 1 diabetes cohorts, multiple islet autoantibodies are strongly associated with accelerated beta-cell decline and insulin dependence [12-15], consistent with the rapid C-peptide decline observed in this patient.

Given the extremely high anti-GAD65 titers characteristic of SPS, prolonged GAD65-directed autoimmunity may facilitate diversification of the autoimmune response toward additional islet antigens such as IA-2 and ZnT8. This may reflect expansion of autoreactive T-cell responses, accelerating beta-cell destruction. Such epitope spreading may represent an immunological link between SPS and rapidly progressive autoimmune diabetes. Because islet autoantibodies can decline after diabetes onset [14], multi-antibody positivity at metabolic decompensation likely reflects earlier diversification, supporting comprehensive autoantibody testing in SPS-associated dysglycemia.

The HLA findings diverge from those reported in European SPS-associated type 1 diabetes cohorts. The canonical susceptibility haplotype DRB1*03:01-DQB1*02:01 [4, 7] was absent, whereas DRB1*04:05-DQA1*03:03-DQB1*04:01, a Japanese susceptibility haplotype for acute-onset type 1 diabetes, was present [8]. The patient also carried DRB1*15:02-DQA1*01:03-DQB1*06:01, confirmed by direct DQA1 typing to carry DQA1*01:03:01 rather than DQA1*01:02, distinguishing it from the Caucasian protective counterpart DRB1*15:01-DQA1*01:02-DQB1*06:02 [10, 11], a haplotype strongly protective against type 1 diabetes in Asian populations [8, 9]. Protective HLA alleles may attenuate susceptibility but do not eliminate risk when coexisting with susceptible haplotypes. Although diabetes was first recognized in 2020, autoimmune type 1 diabetes was established only at the index admission in 2024. This 14-year interval from SPS diagnosis to autoimmune type 1 diabetes confirmation may reflect partial genetic protection insufficient against sustained high-titer GAD65 autoimmunity.

HLA-A*24:02, associated with more complete beta-cell destruction in Japanese patients with type 1 diabetes, was also present [16, 17]. This allele may promote HLA class I–restricted cytotoxic T-cell responses against beta-cell antigens such as preproinsulin [18], potentially contributing to the rapid loss of endogenous insulin secretion. Although antigen-specific T-cell analyses were not performed, this allele may have facilitated accelerated beta-cell destruction once islet autoimmunity expanded beyond GAD65.

An alternative explanation for progressive insulin deficiency is tacrolimus exposure, as calcineurin inhibition can impair insulin transcription and secretory granule formation and may cause hyperglycemia or diabetes [19]. However, tacrolimus-related beta-cell dysfunction is generally considered functional and reversible after discontinuation [20]. A previous report described tacrolimus-induced DKA with recovery of endogenous insulin secretion after discontinuation [21]. In contrast, urinary C-peptide declined from 4.16 μg/day during tacrolimus therapy to <0.9 μg/day at the index admission despite tacrolimus discontinuation, indicating progressive and likely irreversible beta-cell loss.

Progressive beta-cell failure occurred despite prolonged SPS-directed immunotherapy, suggesting that immune mechanisms driving islet autoimmunity may differ from those responsible for neurological manifestations of SPS [22]. Persistent high anti-GAD65 titers further support ongoing autoimmune activity.

This study has several limitations. As a single-case observation, causal relationships cannot be established. Longitudinal measurements of non-GAD islet autoantibodies were unavailable, and differences between assay methods limited direct comparison of anti-GAD65 titers. Antigen-specific T-cell analyses were not performed, precluding assessment of HLA-A*24:02–restricted cytotoxic responses. Larger studies in Asian SPS cohorts are needed to define genotype-specific risk patterns.

## Learning points

- In Japanese patients with SPS, DRB1*04:05 may represent a susceptibility allele for autoimmune type 1 diabetes, contrasting with DRB1*03:01 reported in European cohorts.
- A coexisting protective HLA haplotype may not fully prevent autoimmune diabetes under sustained high-titer anti-GAD65 autoimmunity.
- In SPS patients with worsening glycemia, test for anti-GAD65, anti-IA-2, and anti-ZnT8 to identify triple autoantibody positivity and rapid beta-cell loss.
- SPS-directed immunotherapy does not guarantee beta-cell preservation; urinary C-peptide monitoring is warranted, and insulin therapy should be optimized promptly when beta-cell failure is detected.

## Data Availability

Original data generated and analyzed during this study are included in this published article.

## Abbreviations

ALT: alanine aminotransferase
AST: aspartate aminotransferase
BUN: blood urea nitrogen
CRP: C-reactive protein
DKA: diabetic ketoacidosis
eGFR: estimated glomerular filtration rate
EIA: enzyme immunoassay
HbA1c: hemoglobin A1c
IAP: immunoadsorption plasmapheresis
IVIG: intravenous immunoglobulin
RIA: radioimmunoassay
RBC: red blood cell count
SPS: stiff-person syndrome
WBC: white blood cell count
